# Loss and conservation of evolutionary history in the Mediterranean Basin

**DOI:** 10.1186/s12898-016-0099-3

**Published:** 2016-10-07

**Authors:** S. Veron, P. Clergeau, S. Pavoine

**Affiliations:** Centre d’Ecologie et des Sciences de la Conservation (CESCO UMR7204), Sorbonne Universités, MNHN, CNRS, UPMC, CP51, 43-61 rue Buffon, 75005 Paris, France

**Keywords:** Amphibians, Evolutionary distinctiveness, Endemism, Mammals, Mediterranean basin, Phylogenetic diversity, Protected areas, Squamates

## Abstract

**Background:**

Phylogenetic diversity and evolutionary distinctiveness are highly valuable components of biodiversity, but they are rarely considered in conservation practices. Focusing on a biodiversity hotspot, the Mediterranean Basin, we aimed to identify those areas where evolutionary history is highly threatened and range-restricted in the region. Using null models, we first compared the spatial distributions of three indices: two measured threatened evolutionary history—Expected PD*loss* and Heightened Evolutionary distinctiveness and Global Endangerment—and one measured endemic evolutionary history—Biogeographically Evolutionary Distinctiveness. We focused on three vertebrate groups with high proportions of endemic, threatened species: amphibians, squamates and terrestrial mammals. Second, we estimated the spatial overlap of hotspots of threatened and endemic evolutionary history within the network of protected areas under several conservation scenarios.

**Results:**

Areas that concentrate evolutionary history of conservation interest greatly differed among taxa and indices, although a large proportion of hotspots were identified in the Maghreb, in the East of the Mediterranean Basin as well as in islands. We found that, in a minimum conservation scenario, there was a significant proportion of hotspots for amphibians and squamates that were protected but not for terrestrial mammals. However, in a strong conservation scenario, only few hotspots overlapped with protected areas and they were significantly less protected than in a model where hotspots were chosen randomly.

**Conclusions:**

Some sites concentrate highly threatened and range-restricted evolutionary history of the Mediterranean basin and their conservation could be much improved. These sites are relevant for conservation studies aimed at designing new conservation actions to preserve evolutionary history and the option values it represents.

**Electronic supplementary material:**

The online version of this article (doi:10.1186/s12898-016-0099-3) contains supplementary material, which is available to authorized users.

## Background

Due to human activities, species are going extinct at such high rates that a sixth mass extinction crisis has probably begun [1]. In the future extinction risks are expected to intensify. However, not all species can be saved and conservationists have to make a choice about how to best protect biodiversity [2]. Basing conservation on species richness or threatened species, as is usually the case, may be an inadequate strategy to conserve the diversity of life because it considers all species as equal [3]. A more valuable strategy, in which there is increasing interest, is to protect species evolutionary history. One main benefit of evolutionary history over species richness is that it may capture future diversity and provide future unexpected services for humans and ecosystems [3]. Other benefits are ethical: helping to protect Earth’s evolutionary heritage [4]; aesthetical: humans may appreciate a variety of living forms [5]; and evolutionary: providing possibilities for future evolution [6] (but see [7]). Loss of evolutionary history could be much higher than species richness loss when extinctions are phylogenetically clustered (thus threatening not only terminal branches but also deep branches shared by the species at risk), when evolutionarily distinct species go extinct and when the phylogenetic tree is unbalanced, i.e. the extent to which some branches lead to many species (or higher taxa) while their sister branches lead only to a few [8–10]. However, evolutionary history is rarely included in conservation programs and is poorly represented in protected areas [10]. Depending on those factors, evolutionary history is more threatened in some regions of the world than in others [10]. In this study, we are interested in the risks of losing evolutionary history in the Mediterranean Basin, one of the richest regions of biodiversity on Earth and where many endemic and threatened species live.

The Mediterranean Basin is situated at the junction of Europe, Africa and Asia. It extends eastward from Morocco to Turkey and southward from northern Italy to the Canary Islands. Countries of the Mediterranean Basin share a common climate [11], which is characterized by hot, dry summers and cool, humid winters. This climate strongly influences the wildlife of the Mediterranean Basin such that many species are found nowhere else on Earth [12]. Moreover, intense human activity has resulted in landscapes characterized by complex patchworks of habitats, generating a high diversity of species [11]. The Mediterranean Basin has been identified as a hotspot in terms of its diversity and its high ratio of endemism in plants [13, 14]. It also shelters a rich but threatened diversity of marine and terrestrial animals that includes many endemic species [15]. Among Mediterranean species, 26 % of mammals, 48 % of squamates and 64 % of amphibians are endemic to the region [11].

The degradation of habitats, climate change, invasive species, overexploitation of natural resources and pollution are the most significant threats to biodiversity in the region, causing extensive damage to ecosystems, fauna and flora [11]. These threats, particularly habitat degradation and climate change, are expected to intensify in the future [15]. Moreover the recent growth of tourism activities increases the risks of losing biodiversity [16]. Of the 1912 species evaluated by the International Union for the Conservation of Nature (IUCN) in the Mediterranean region (including amphibians, squamates, birds, mammals, crayfish and crabs, cartilaginous fishes, endemic fresh water fishes and dragonflies), 19 % are threatened with extinction [11].

The IUCN has analysed the threats to biodiversity in the Mediterranean Basin and advocated the protection of those endemic species that capture unique phylogenetic information [11]. To date, few studies have considered evolutionary history or its conservation in the region, although several studies have explored fish evolutionary history [17, 18]. A valuable strategy to measure the evolutionary history of conservation interest is to use phylogenetic diversity (PD) and evolutionary distinctiveness (ED) metrics. The PD of a subset of taxa is measured as the sum of the branch lengths of the minimum path that joins those taxa on a phylogenetic tree [19]. ED quantifies the number of relatives a species has and how phylogenetically distant they are [19, 20]. PD and ED are complementary measures for conservation. PD identifies the amount of shared evolutionary history of the species present in an area and may capture functional diversity and future benefits [3, 19, 21]. ED enables us to prioritize species according to their phylogenetic isolation (which decreases with the number of relatives and increases with the phylogenetic distance to those relatives) and may capture rare features important for ecosystem services [22, 23]. Preserving ED species may also help to conserve PD when all species which maximize PD cannot be protected [24]. Due to the high rates of endemism and the threats faced by species in the Mediterranean basin, our objectives were to identify those terrestrial areas where PD and ED are threatened or range-restricted and to analyse the effectiveness of protected areas in conserving those hotspots. To match with conservation policies we identified hotspots according to the Aïchi target defined by the Convention on Biological Diversity in 2011 [25] which aims to protect 17 % of land areas. We considered protected areas in categories I, II, and IV as their main management objective is to directly protect species [26]. We focused on the vertebrate groups that are known to have high proportions of endemic and threatened species in the region: amphibians, squamates and mammals.

## Results

We searched for hotspots that concentrate threatened evolutionary history according to three indices:

1. Expected PD*loss* [27], which calculates at-risk PD: branch lengths of the phylogeny are weighted by the extinction probabilities of the species they support and Expected PD*loss* is the sum of those weighted branches. The use of this metric was highly recommended to measure the total branch length at risk because it accounts for the phylogenetic complementarities of extinction risks, i.e. the extinction probability of a deep branch depends on the probability that all its descendant species go extinct. Expected PD*loss* identifies the amount of threatened evolutionary history of the species present in an area.

2. Heightened Evolutionary Distinctiveness and Global Endangerment (HEDGE) [28], which calculates at-risk ED in a probabilistic framework where the branch lengths of the phylogeny are also weighted by the extinction probabilities of the species they support. Similarly to Expected PD*loss*, its use was recommended because it is based on the phylogenetic complementarities of extinction risks. However, contrary to Expected PD*loss*, it gives a score to each species.

3. Biogeographically weighted Evolutionary Distinctiveness (BED) [29], which identifies species that encompass high amounts of ED and are also the most spatially restricted. Like HEDGE, it considers phylogenetic complementarity (but with range sizes instead of extinction probabilities) and it gives a score to each species.

### Hotspots depend on indices and taxa

For mammals, 24 % of the hotspots were identified by all three indices, whereas 22 and 15 % of hotpots were shared by the three indices in squamates and amphibians, respectively.

**Fig. 1:**
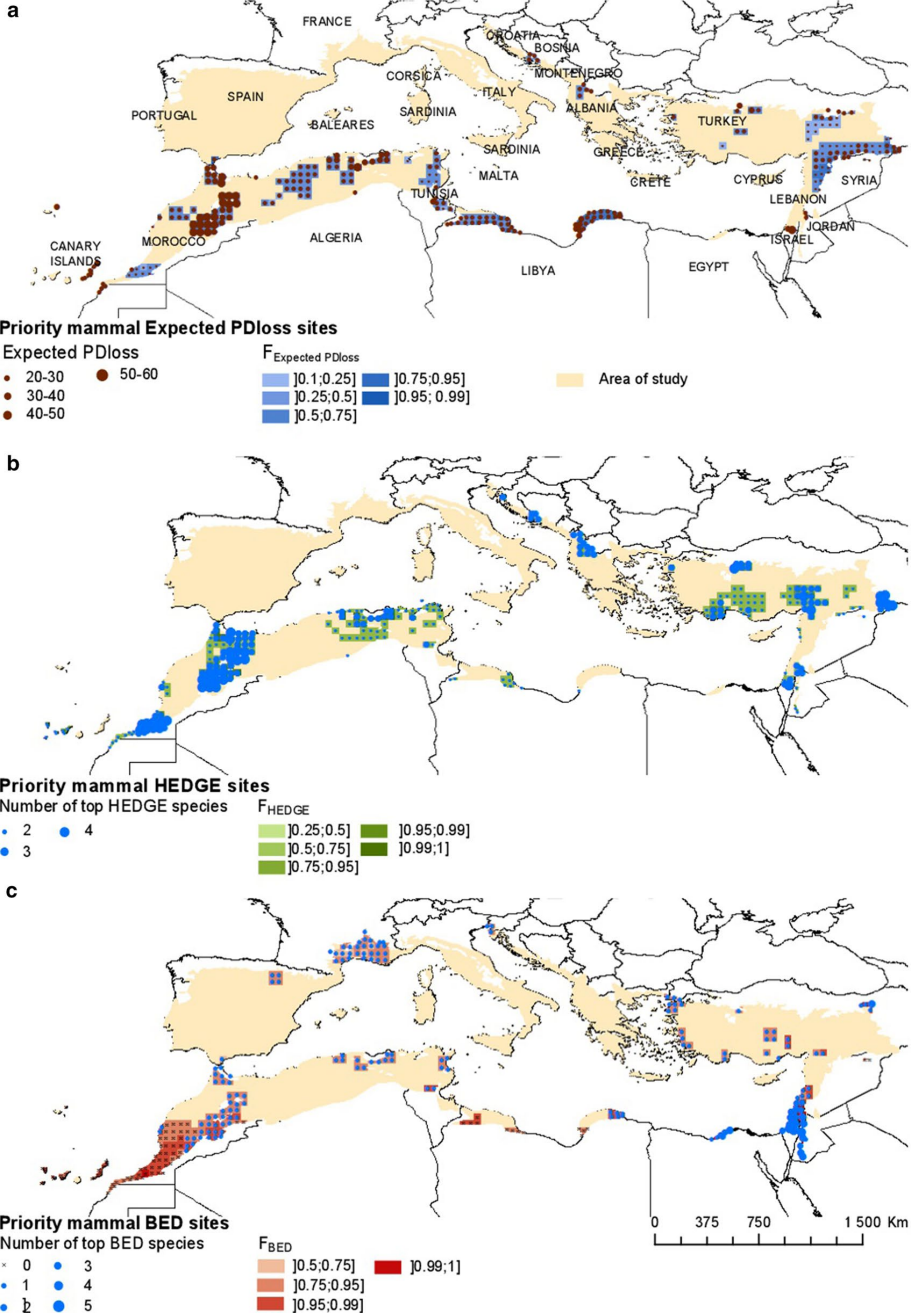
Spatial distribution of hotspots of threatened evolutionary history in mammals **a** Expected PD*loss* **b** HEDGE and **c** BED. Hotspots were selected according to their high F_Expected PDloss_, F_HEDGE_, F_BED_ values, with ties discriminated according to raw Expected PD*loss*, HEDGE and BED, respectively as detailed in the main text. To represent F_Expected PDloss_, F_HEDGE_, and F_BED_ categories we used half-closed intervals in order to avoid any overlap between them, excluding the first value of each interval

For mammals, considering the Aïchi target of 17 % of protected territory, the areas that were expected to lose disproportionate amounts of PD (according to expected PD) and which harbour top HEDGE species were situated in the Maghreb, Turkey, the Balkans, Israel and the Canary islands (Fig. 1a, b). Some key BED areas were found in southern Morocco, Israel, Lebanon, Jordan, northern Egypt, Turkey and southern France (Fig. 1c). For example, *Macaca sylvanus*, the endangered and only primate of the Mediterranean region, is found only in northern Morocco and Algeria. The Equidae species *Equus hemonius* is also highly threatened, with only one small, reintroduced population in Israel (Fig. 4a).

**Fig. 2:**
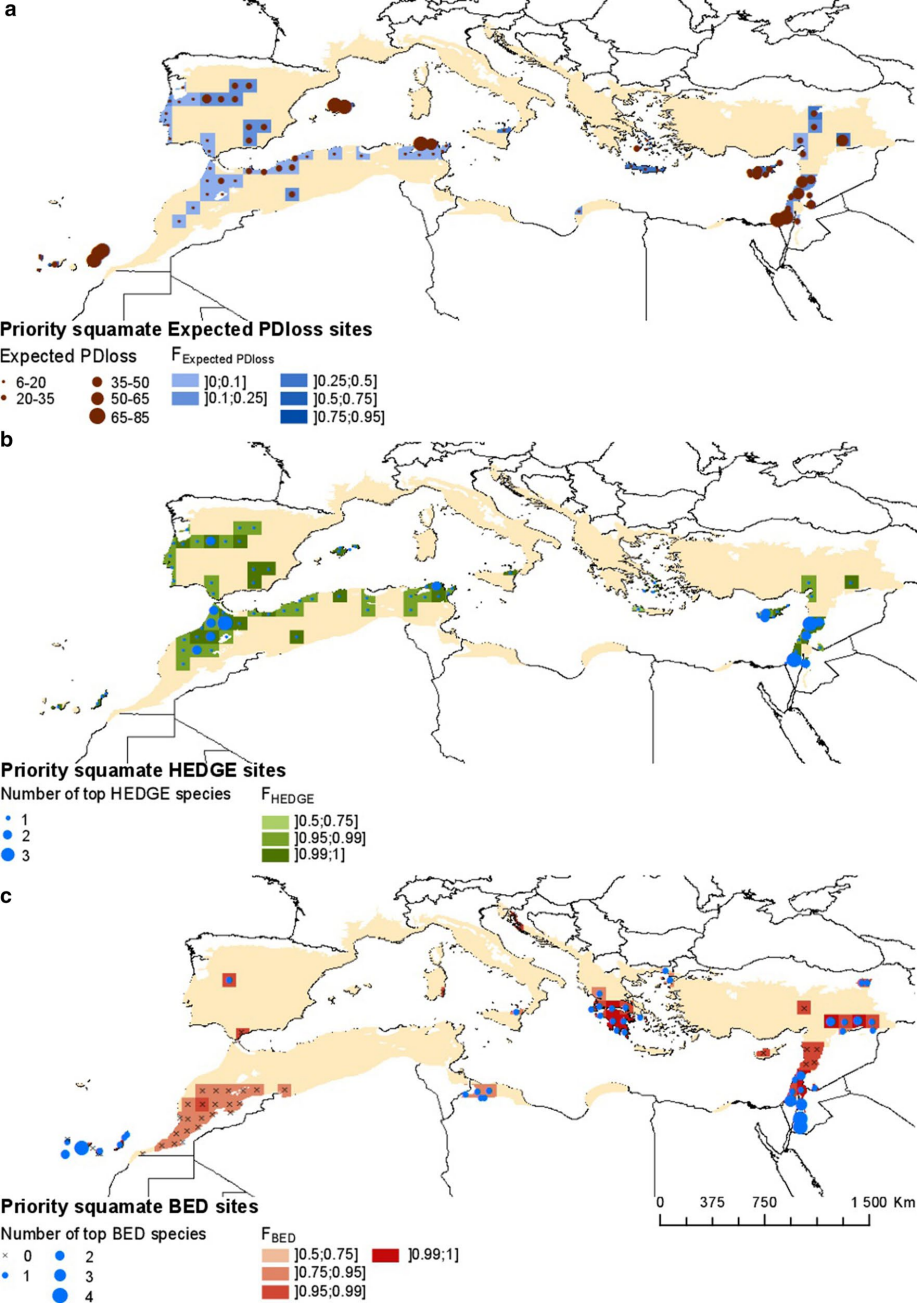
Spatial distribution of hotspots of threatened evolutionary history in squamates **a** Expected PD*loss* **b** HEDGE and **c** BED. Hotspots were selected according to their high F_Expected PDloss_, F_HEDGE_, F_BED_ values, with ties discriminated according to raw Expected PD*loss*, HEDGE and BED, respectively as detailed in the main text

For squamates, the hotspots based on Expected PD*loss* and HEDGE were identified in Israel, Lebanon, central Spain, northern Maghreb and islands: the Baleares, Canary Islands, Crete, Cyprus, and north of Sicily (Fig. 2a, b). Key BED sites were mainly located in the Canary Islands, Israel, Lebanon, Jordan, and Greece (Fig. 2c). Several top HEDGE species were highly restricted and had high BED scores (Fig. 4b). In particular, the lizard genus *Ibolacerta* represents an important evolutionary radiation in the Mediterranean Basin, with a high proportion of endemic and threatened species.

**Fig. 3:**
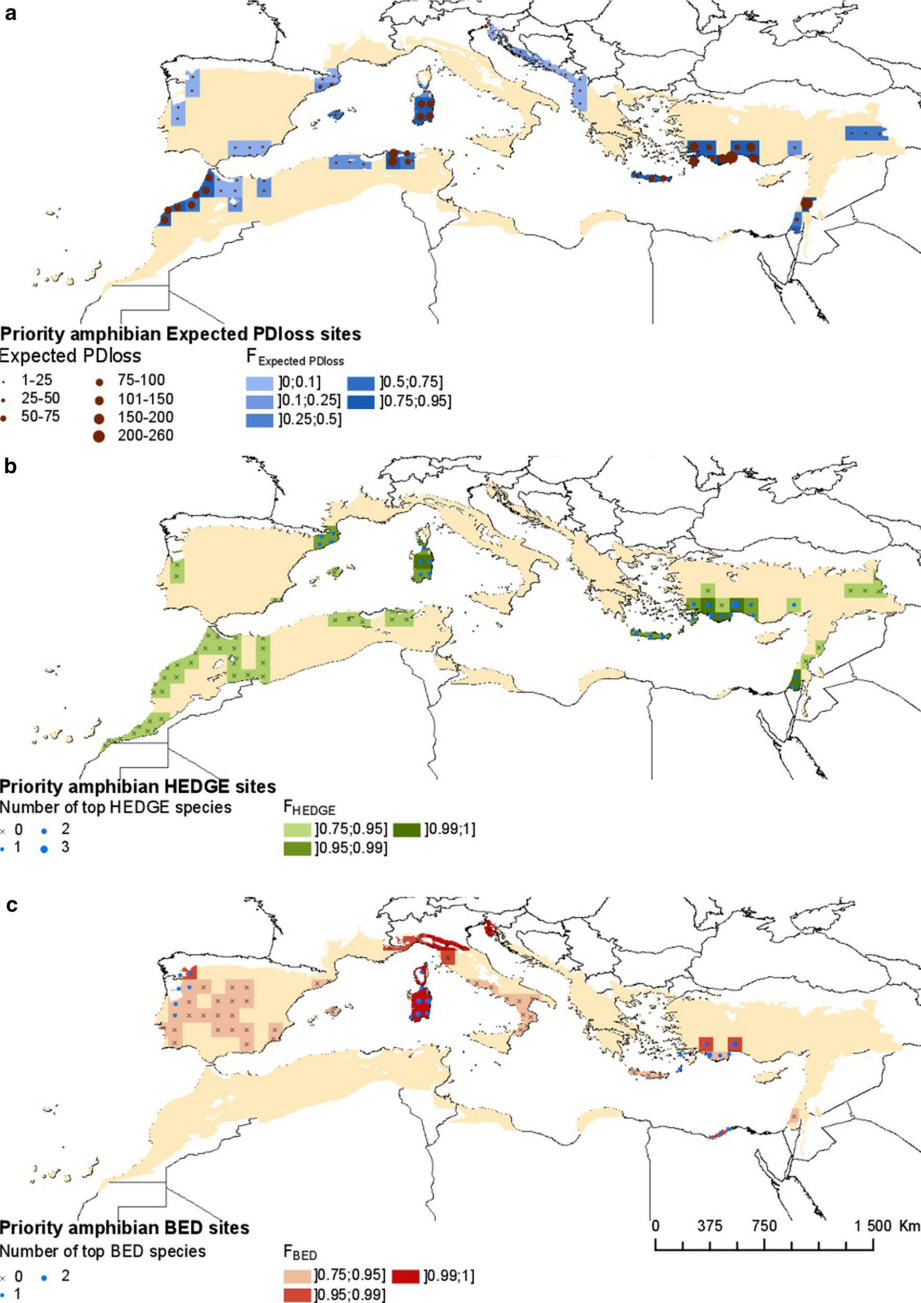
Spatial distribution of hotspots of threatened evolutionary history in amphibians **a** Expected PD*loss* **b** HEDGE and **c** BED. Hotspots were selected according to their high F_Expected PDloss_, F_HEDGE_, F_BED_ values, with ties discriminated according to raw Expected PD*loss*, HEDGE and BED, respectively as detailed in the main text

**Fig. 4:**
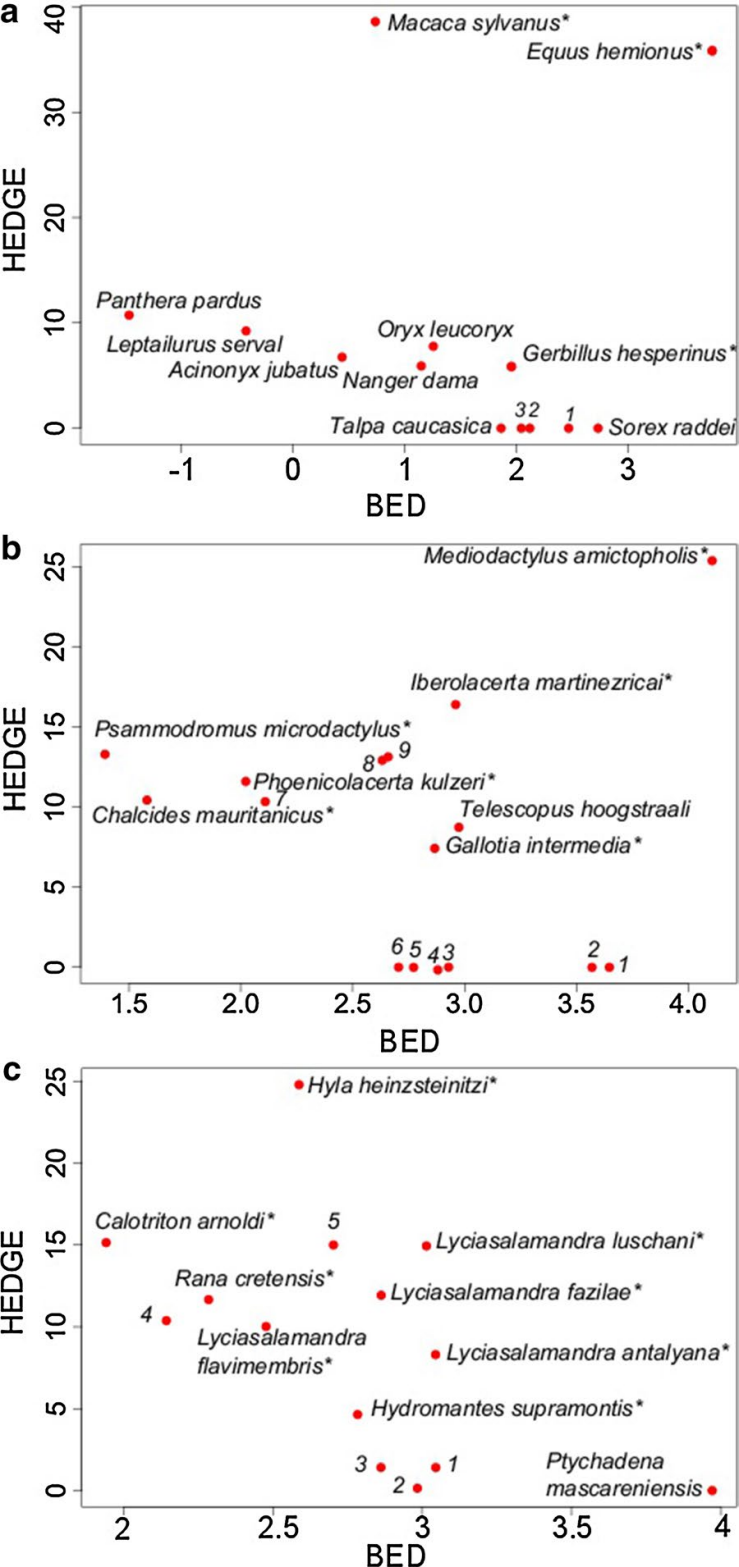
Top HEDGE and BED species. The *graph* represents top heightened evolutionary distinctiveness globally endangered (HEDGE) scores against top biogeographically evolutionary distinctiveness (BED) scores for squamate, amphibian and terrestrial mammal species. BED scores are represented with a logarithm scale. A *means that the species is endemic from the Mediterranean Basin. **a** Top ten HEDGE and top ten BED mammal species *1 Gerbillus floweri*, *2 Gerbillus cheesmani*, *3 Arvicanthis niloticus*. **b** Top 10 % HEDGE and top 10 % BED squamate species. 1 Pristurus rupestris 2 Platyceps sinai 3 Asaccus elisae 4 Tarentola gomerensis* 5 Bunopus tuberculatus 6 Acanthodactylus tilburyi 7 Algyroides marchi* 8 Podarcis raffonei* 9 Acanthodactylus beershebensis* **c** Top 10 % HEDGE and top 10 % BED amphibian species. 1 Hydromantes genei* 2 Discoglossus montalentii* 3 Lyciasalamandra helverseni* 4 Euproctus platycephalus* 5 Lyciasalamandra billae*

For amphibians, HEDGE, Expected PD*loss* and BED hotspots were identified in Sardinia, the Spanish Pyrenees, Morocco, northern Algeria, the Balkans, Crete and southern Turkey (Fig. 3a–c). Some species, in particular those from the genus *Lyciasalamandra* and the critically endangered *Hyla heinzsteinitzi*, ranked high in both BED and HEDGE scores (Fig. 4c).

For all groups, the identified hotspots captured high proportions of the regional Expected PD*loss* as well as high proportions of accumulated regional HEDGE and BED values (sum of species HEDGE and BED values). These proportions were significantly higher than the proportions obtained when hotspots were chosen randomly: all p values < 0.01 except HEDGE and Expected PD*loss* for mammals (p < 0.1) (Table 1).

**Table 1:** Unique threatened and endemic evolutionary history represented in priority grid cells

	Terrestrial mammals	Squamates	Amphibians
Expected PD*loss*	88.59*	97.18**	81.7**
HEDGE	90.96*	94.87**	87.16**
BED	95.71**	86.92**	73.15**

The table represents the proportion of unique Expected PD*loss*, HEDGE and BED captured by the corresponding hotspots. To calculate p values we determined how often the proportion of unique Expected PD*loss*, HEDGE and BED, captured by a random selection of hotspots, was higher than or equal to the observed values

Significance represented with the symbol * corresponds to marginal significance (p ≤ 0.1) and ** to significance (p ≤ 0.01)

When choosing hotspots independently of the 17 % Aïchi threshold, we identified fewer hotspots than 17 % of total cells, except for mammal HEDGE hotspots which, in that case, covered more than 17 % of the land (Additional files 1, 2, 3). In mammals, many additional hotspots with high HEDGE values were found in the North of Maghreb and in Turkey (Additional file 1).

We found several moderate correlations between the distribution of species richness and PD, Expected PD*loss*, HEDGE and BED values (Table 2; see also maps of species richness in Additional files 4, 5, 6). Places where species are highly threatened are likely to be hotspots of evolutionary history at risk. Especially in amphibians, sites in southern Turkey, in Israel and in Sardinia have many threatened species and are hotspots for Expected PD*loss*, HEDGE and BED. Yet a species richness approach also misses some sites where threatened evolutionary history concentrate. For example, the Canary Islands are hotspots of terrestrial mammal Expected PD*loss* although relatively few threatened species are found there (Fig. 1; Additional file 4).

**Table 2:** Correlations between a phylogenetic and a species richness approach

	Terrestrial mammals	Squamates	Amphibians
Correlations with species richness			
PD	0.88^**^	0.94^**^	0.95^**^
Correlations with richness in threatened species			
Expected PD*loss*	0.65^**^	0.47^**^	0.68^**^
HEDGE	0. 70^**^	0.38^**^	0.71^**^
Correlations with richness in range-restricted species			
BED	0.37^**^	0.46^**^	0.27^**^

Spearman correlation between evolutionary history and species richness for PD, richness in threatened species (species classified as critically endangered, endangered, or vulnerable) for Expected PD*loss* and HEDGE and richness in range-restricted species (number of species in the top 10 % species with the smallest range size) for BED

Significance represented with the symbol * corresponds to marginal significance (p value ≤ 0.1) and ** to significance (p value ≤ 0.01)

### Poorly protected hotspots

**Fig. 5:**
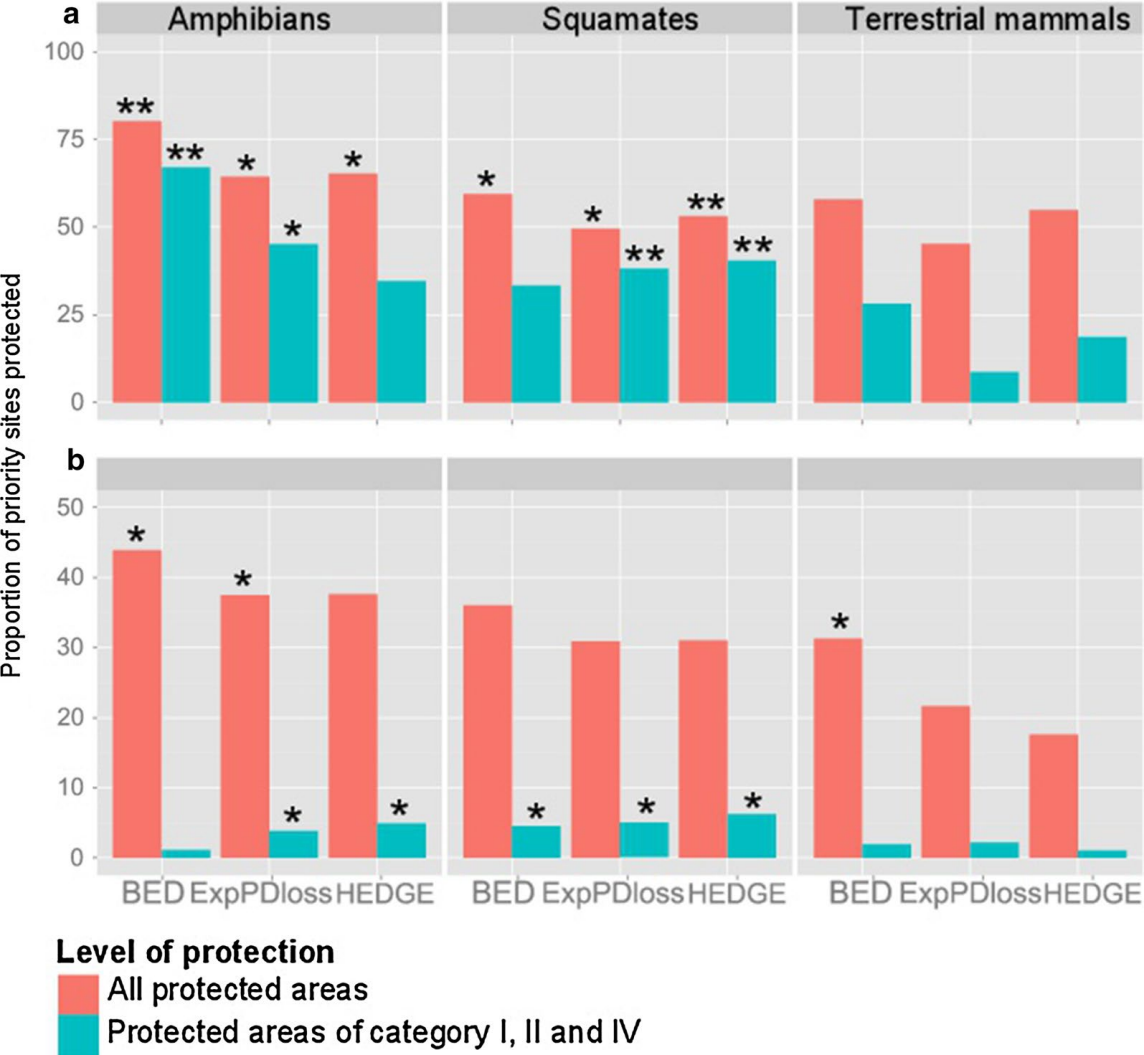
Proportion of hotspots of threatened and endemic evolutionary history protected in two conservation scenarios. Each graph represents the proportion of HEDGE, BED and Expected PD*loss* hotspots protected. Red bars correspond to the degree of protection if all protected areas are included and blue bars the degree of protection if only protected areas of categories I, II and IV are included. Star symbols correspond to the frequency to which the proportion of hotspots protected was higher than if priority grid cells were distributed randomly (F_PA_): no star means F_PA_ ≤ 0.25; * means 0.25 < F_PA_ ≤ 0.75; **0.75 < F_PA_ ≤ 1. We ran analysis for **a** a scenario of minimum protection in which hotspots were safe if they intersected at least one protected area; **b** a scenario of strong protection where a site was considered safe if it was protected on more than half of its area

The number of hotspots that overlapped with protected areas varied among groups. Nonetheless, the degree of protection was low for all groups; i.e. only a few hotspots were protected on more than half of their surface, particularly in the categories I, II and IV of protected areas (Fig. 5).

### Coverage by all Mediterranean protected areas

In the minimum protection scenario for mammals, approximately 60 % of BED hotspots intersected with at least one protected area; however, these sites were significantly under-protected (significance refers to the frequency to which the proportion of hotspots protected was higher than if priority grid cells were distributed randomly (F_PA_): significantly under-protected means F_PA_ ≤ 0.25, significantly over-protected means 0.75 < F_PA_ ≤ 1; Fig. 5a). Both Expected PD*loss* and HEDGE mammal hotspots were significantly under-protected, and 45 and 55 % of these sites, respectively, contained at least one protected area (Fig. 5a). Around 50 % of squamate hotspots intersected with at least one protected area for all indices (Fig. 5a). These proportions were similar to those expected if hotspots were randomly distributed for BED and Expected PD*loss* indices and greater than expected for the HEDGE index (Fig. 5a). For amphibians, 65 % of HEDGE and Expected PD*loss* hotspots intersected with protected areas and they were as protected as expected from a random distribution of hotspots. BED hotspots were significantly over-protected and 80 % of them intersected with at least one protected area (Fig. 5a).

However, in the strong protection scenario, where sites were considered protected when more than half of their area was covered by protected areas, the proportion of protected hotspots decreased (Fig. 5b). For terrestrial mammals, hotspots were significantly under-protected for Expected PD*loss* and HEDGE indices but as protected as expected from random for BED hotspots (Fig. 5b). In amphibians, HEDGE hotspots were significantly under-protected whereas BED and Expected PD*loss* hotspots were as protected as expected from random (Fig. 5b). In squamates hotspots were significantly under-protected for all indices (Fig. 5b).

### Coverage by Mediterranean protected areas of category I, II and IV

We repeated the same analyses with only the categories I, II and IV of protected areas, i.e., protected areas specifically dedicated to species conservation or with stringent regulations. As expected, for all groups, the proportion of protected hotspots decreased compared with that observed where all protected areas were considered (Fig. 5). In amphibians, protected areas of category I, II and IV overlapped with as many Expected PD*loss* hotspots as expected from random. Amphibian BED hotspots were significantly over-protected but HEDGE hotspots were significantly under-protected (Fig. 5a). In squamates only BED hotspots were significantly under-protected whereas, in terrestrial mammals, hotspots were significantly under-protected according to all indices. Very few sites had more than half of their area covered by protected areas of categories I, II and IV; nonetheless, there were as many such sites as under a random distribution of hotspots in squamates and amphibians, except for amphibian BED hotspots, whereas in mammals BED, HEDGE and Expected PD*loss* hotspots were significantly under-protected (Fig. 5b). Some species with high BED and HEDGE scores were indeed not found in any protected area (Additional file 9). In mammals 13 and 2 species from the top 10 % BED (28 species) and HEDGE species (23 species), respectively, were not found in any protected area. In squamates, 8 and 5 species from the top 10 % BED (24 species) and HEDGE species (23 species), respectively were not found in any protected area. As for amphibians, 3 of the top 10 % BED species (11 species) and 4 of the top 10 % HEDGE species (11 species) were not found in protected areas (Additional file 10).

When hotspots were not defined according to Aïchi targets, hotspots were globally over protected compared to a random arrangement of protected areas for squamates, whereas hotspots for amphibians were as protected as expected from random and hotspots for mammals were significantly under-protected. In a scenario where a higher degree of protection was required, only few hotspots were covered on more than half of their surface by protected areas of category I, II or IV and they were as protected or less protected than random (Additional file 7).

## Discussion

Localization of hotspots differed between taxa but many were identified in the Maghreb, in eastern countries (Israel, Lebanon, Jordan and Turkey) and in islands. For mammals, 24 % of hotspots were common to the three indices, whereas 22 and 15 % of hotpots were shared by the three indices in squamates and amphibians, respectively. Differences between groups may be due to a higher number of top HEDGE species which are also in the top BED species in mammals and to the narrower distribution of threatened and/or endemic amphibians with high evolutionary distinctiveness. These top hotspots supported by all indices revealed areas that capture both high amounts of threatened and range-restricted evolutionary history. For example, in amphibians, some sites in southern Turkey and in Israel were identified as hotspots for all indices and harbour top HEDGE species which also had high BED scores, e.g. *Lyciasalamandra billae*, *Hyla heinzsteinitzi* (Fig. 4; but see the discussion about the taxonomic uncertainty of *Hyla heinzsteinitzi* [30]). Recently, *L. billae* was also identified as one of the top 15 vertebrate species with the highest probability of extinction [31], indicating the importance of conservation efforts in the Mediterranean Basin. However, this overlapping of priority zones did not always occur, and each index provided unique information emphasizing particular conservation requirements. We also found several moderate correlations between the distribution of species richness and Expected PD*loss*, HEDGE and BED values, challenging the use of surrogates among indices [32]. PD and species richness distribution are expected to be, at least partially, correlated (Table 2). However, they are also expected to differ under some conditions, including when phylogenies are unbalanced, closely related species tend to be found near to each other, old species have smaller geographical distributions on average than young species, and old species are found in species-poor areas [33]. The correlation with a species richness approach was lower for the indices we used (Expected PD*loss*, HEDGE and BED) than for PD probably because these indices include information about threat status or range size of deep branches based on phylogenetic complementarity, i.e. the fact that the risk to lose a deep branch depends on the probabilities of extinctions of all the species it supports. For example, even if a site is occupied by a threatened species, deep phylogenetic branches can be secured if non threatened descendants of these branches also live there. As for BED the range size of a deep branch depends on the size of the union of the range of the species it supports.

Our method was based on a traditional, widely used hotspot approach [14, 34–36]. Our aim was to identify areas that contain disproportionate amounts of threatened and endemic evolutionary history, even if some threatened and endemic branches may be present in several hotspots [37, 38]. Similarly, several priority hotspots for the conservation of the evolutionary history of marine mammals at risk were identified in the Mediterranean sea [38]. An alternative to the hotspot approach is the network approach that specifically analyses how many species or how much evolutionary history is shared by sites. The network approach searches for a minimum set of areas that capture as many species or as much evolutionary history in a region as possible [39, 40]. The network approach is of particular interest at a regional scale but we believe the hotspot approach enables us to identify sites whose ecosystem resilience may be threatened and where “option values”, captured by evolutionary history, are at risk [41, 42]. Option values are the values of preserving the option to use services in the future [25]. They are wholly unanticipated and because evolutionary history is expected to capture genotypic, phenotypic and functional diversity it may be the best measure to preserve those as-yet-unexpected services and to promote system resilience in a changing world [27, 43]. An advantage of the hotspot approach is thus to identify sites where option values are at risk at a local scale whereas a network approach would have prioritized sites relevant for conservation at the regional scale. Regional and local conservation needs thus differ, yet local hotspots also contain a very high proportion of Mediterranean threatened and endemic evolutionary history especially within the squamates and amphibians (as shown in Table 1). At the species level, evolutionary distinctiveness may also capture unique features and current and future benefits [44, 45]. Species with high HEDGE and BED values are thus of conservation interest because they may represent at risk option values (Additional file 11). Mouillot et al. [46] showed that rare species represent a large proportion of unique feature diversity which will potentially help to maintain ecosystems that are resilient to threats such as climate change. By capturing rarity and evolutionary history, BED species may thus be key species for the preservation of option values. In this study, we used regional and global data on extinction risks. When available for future studies, adding information about local threat would enable us to refine the definition of Mediterranean hotspots. Moreover an increasing knowledge on the biodiversity of the region and the range distribution of species will improve our comprehension of the risks to lose phylogenetic diversity. Especially, in the future, initiatives such as the global assessment of reptile distribution [47] will help to have more data on the distribution of squamates which were missing in our study. Yet because of the threats, endemism and evolutionary distinctiveness of the species already present in our data we believe the hotspots we identified are important areas for conservation and that more data will contribute to the identification of new hotspots.

Our initial results suggested that hotspots were well covered by protected areas, especially for squamates and amphibians. However, these results were based on the inclusion of all categories of protected areas and the consideration of a cell as protected if it intersected at least one protected area. A more stringent conservation strategy would require greater coverage of sites by protected areas and include management objectives explicitly directed toward species conservation. When such criteria were accounted for, the number of hotspots protected was low. In addition, some species ranking among the species with the highest BED and HEDGE scores were not found in any protected area (e.g. *Gerbillus hesperinus* in mammals, *Acanthodactylus harranensis* in squamates or *Lyciasalamandra antalyana* in amphibians). This gap can potentially be explained by the low coverage of the land by protected areas (4.3 %) [48], more common networks of protected areas in the North such as Natura 2000, lack of protected areas in arid zones (whereas nearly 400 sites have been designed as RAMSAR sites for the protection of wetlands [49]) and more numerous protected areas dedicated to bird conservation (e.g. Bird Directive 2009/147/EC).

Previous studies have highlighted the poor conservation of evolutionary history. At a global scale, Safi et al. [34] found that only 15.6 and 4.7 % of evolutionary distinct and globally endangered amphibian and mammal priority zones, respectively, intersected with protected areas. In Europe, terrestrial mammal, squamate and bird PD and ED are less protected than expected if protected areas were randomly distributed [50, 51]. At the Mediterranean scale, Guilhaumon et al. [18] showed that protected areas in the Mediterranean Sea did not reach conservation targets for fish PD (see also [17]). An approach to conserving Mediterranean PD and ED would be to consider evolutionary history in the definition of Key Biodiversity Areas [52]. Key Biodiversity Areas are sites of global significance for biodiversity conservation and are identified using standard criteria such as vulnerability and irreplaceability. They are a basis for conservation planning and are important to the maintenance of viable species populations [53]. Evolutionary history criteria are not yet included in the standards that define Key Biodiversity Areas, but Brooks et al. [54] proposed the inclusion of at-risk phylogenetic endemism and evolutionary distinctiveness in the standards. By measuring the spatial distribution of BED, Expected PD*loss* and HEDGE, we identified hotspots that may inform the establishment of new Key Biodiversity Areas. However, further research is needed to develop an approach that combines the advantages of the hotspot and of the network approaches by considering local and regional conservation needs while also considering other essential principles for reserve design, such as costs, flexibility and irreplaceability. It would also be meaningful to estimate the coverage of hotspots with already defined Key Biodiversity Areas. Another possible measure to make the conservation of PD more stringent would be to dedicate some already protected areas specifically to the conservation of species and of their evolutionary history. Indeed, some hotspots exclusively contain protected areas of category III, V and VI, which do not directly aim to conserve species. For example the management objective of protected areas classified under the category V is to protect landscapes. Conservation areas are not the only way to conserve biodiversity; many species exist beyond protected areas, and a key goal should be to preserve the quality of their habitats [31]. Evolutionary history criteria should also be included in prioritization lists of species [55]. This may be an important complementary approach to species conservation, as conservation objectives within protected areas are not always met [56].

## Conclusion

We conducted the first study that identifies those areas where at-risk and range-restricted evolutionary history is concentrated in the Mediterranean Basin. Hotspots were mainly found south and east of the Mediterranean Sea and in islands but were poorly covered by protected areas. We also showed that some species representing the threatened endemic evolutionary history of the region were not found in any protected area. Thus, not only local sites but the region itself are at risk of losing large amounts of phylogenetic diversity. Underwood et al. [57] stressed the importance of expanding the network of Mediterranean protected areas; we showed that new protected areas should be delineated to avoid that some sites lose too much of their phylogenetic diversity. We thus encourage practitioners to consider evolutionary history criteria in their efforts to protect habitats, ecosystems, species and their related benefits to societies. We recommend to use both a site and a species approach and not only to consider species threat status but also endemism, while accounting for the different categories of protected areas. We advise the use of indices which consider the phylogenetic complementarities of extinction risks or range restrictions, meaning that a branch in the phylogeny may be lost only if all its descending species are lost and that it is range-restricted only if its descending species are all endemic to the same restricted area. We also encourage the use of null models, as they enable us to identify sites at risk independently of species richness. As the resolution and completeness of phylogenies improve, the use of phylogenetic diversity in conservation is becoming increasingly reliable and meaningful. Future assessments of conservation needs at different scales as well as assessments of data-deficient species status could enhance our knowledge of the risks of losing evolutionary history.

## Methods

### Data

For the mammal phylogeny, we used a maximum clade credibility tree [58]. We used recently established phylogenies for squamates [59] and amphibians [60], both phylogenies being fully resolved and dated. Yet, some Mediterranean amphibian and squamate species were not present in those phylogenies. Some of them were highly threatened and/or endemic of the basin and could represent evolutionary history of conservation interest (3 mammals, 1 squamate and 4 amphibians among missing species were threatened and 8 mammals, 4 squamates, 6 amphibians missing in the phylogenies were endemic to the region) we thus included them by creating polytomies with species belonging to the same genus. Fifteen mammals, 15 squamates and 5 amphibians were added as polytomies. It was shown that the effect of polytomies on PD and ED may be very low [10]. To test whether the placement of missing species had little impact on the identification of hotspots we randomized all species for each genus in the phylogenies and found that observed HEDGE, Expected PD*loss* and BED rankings of grid cells were highly correlated with rankings obtained under the randomization procedure (Spearman correlation test; rho > 0.95 and p ≪ 0.001 for all indices)

Spatial data were mapped using ArcGIS 9.3 software. We used the ecoregion limits of the Mediterranean Basin [61]. We have delimited the Mediterranean Basin as the biome “Mediterranean forests, woodlands, and scrub” of the Palearctic Realm. Distribution ranges of species were downloaded from the IUCN extent of occurrence maps [62]. All maps were projected in a World Behrman projection.

The conservation status of each species was downloaded from the IUCN Red List assessments [62]. We used the regional assessments for terrestrial mammals [63] but used the global assessments for amphibians and squamates because no Mediterranean assessments exist for these two groups [62]. We removed data-deficient (DD) species to calculate the HEDGE and Expected PD*loss* indices but not the BED index, as this latter index does not rely on threat categories (see the next section for a definition of these indices). For all indices, we removed extinct and regionally extinct species, and in mammals, we removed those species for which a regional assessment was not applicable. Our final data set comprised 229 terrestrial mammals, 107 amphibians and 230 squamates for HEDGE and Expected PD*loss* and 258 terrestrial mammals, 107 amphibians, and 238 squamates for BED; higher numbers for BED were due to the integration of species classified data-deficient in the IUCN Red List (Additional file 10). All known Mediterranean terrestrial mammals and amphibians were included whereas some information about the distribution of squamates was missing (342 squamates were assessed in the region; [62]). More justification on the data used can be found in the Additional file 8.

Protected areas were downloaded from the most recent updates of the world database on protected areas [64]. Some shape information was missing for some protected areas; these areas were thus represented as points. We added buffer areas around these points that corresponded to the respective sizes of the areas. Protected areas for which both shape and size data were missing (24 protected areas) were excluded from analysis. The final data set included 9093 protected areas.

### Metrics

We first assessed Expected PD*loss* [27] and HEDGE metrics [28], using probabilities of extinction within 50 years based on the transformation of the IUCN categories into extinction probabilities as described by [65]. We then calculated BED from the range size of species [29]. We used R [66] and the most recent versions of the picante [67] and phylobase [68] packages for analysis.

### Identifying hotspots where evolutionary history is at risk

We first defined and mapped hotspots of Expected PD*loss*, HEDGE and BED values according to the following procedure. We applied a 1° × 1° resolution grid to the Mediterranean Basin map for squamate and amphibian analyses (corresponding to approximately 100 × 100 km and 477 grid cells) and 0.5° × 0.5° for mammal analyses (50 km × 50 km and 1489 grid cells). We made this choice as a compromise between having a sufficient number of species per grid cell, having an accurate resolution and decreasing omission errors. Moreover it was shown that, for mammals, at a 0.5° × 0.5° resolution distribution data from the IUCN and atlas data were similar [69] showing the reliability of data at this scale. We made a complementary analysis at a 1° × 1° resolution for mammals and found that hotspots were identified in the same region and the proportion protected was similar to the results found at a 0.5° × 0.5° resolution (unpublished result). Similarly Safi et al. [34] found that the resolution of grid cells did not affect the identification of sites where threatened evolutionary history concentrate. We then calculated the Expected PD*loss* value, the number of top-priority HEDGE species, and the number of top-priority BED species for each cell and species group.

We defined “top” species as the 10 % of species with the highest scores for a given index (either HEDGE or BED). This approach depends on the number of species in each cell (Table 2; see also [34]). We thus defined a second criterion to identify hotspots by using null models as follows.

For Expected PD*loss*, we identified those areas with greater losses than expected if extinction risks were randomly distributed. We randomized extinction risks among species in the phylogeny one thousand times, calculated the new value of Expected PD*loss* in each grid cell for each randomization, and then determined the frequency (termed F_Expected PDloss_) with which the observed Expected PD*loss* value in a given grid cell was higher than the simulated values. For the HEDGE and BED values, we randomized species identities within each grid cell one thousand times while maintaining the species richness of each grid cell constant. We then calculated the sum of species HEDGE and BED values per grid cell for each randomization and calculated the F_HEDGE_ and F_BED_ values as the frequencies with which the observed HEDGE and BED values in a given grid cell were higher than the simulated values. We thus used two classes of null models. Because Expected PD*loss* is measured as a characteristic of a given site, we fixed the composition of each cell and randomized species extinction risks. In contrast, because HEDGE and BED values are measured as characteristics of each species independently of the cell in which they occur, we shuffled species across cells. The two approaches thus complement each other to reveal hotspots for conservation.

Hotspots were identified based on Aïchi targets defined in the Strategic Plan for Biodiversity 2011–2020 and aiming to protect 17 % of the total land area [25]. We ranked grid cells in increasing order of F_Expected PDloss_, F_HEDGE_ and F_BED_. Ranks for ties were determined using the raw Expected PD*loss*, HEDGE or BED values. For example, grid cells with equal F_Expected PDloss_ values were ranked in increasing order of Expected PD*loss*. Then, we selected the 17 % of grid cells with the highest ranks. We also proposed an alternative selection of hotspots independent of Aïchi targets in Additional files 1, 2, 3 and 7. This alternative strategy enables to identify either the most threatened and range-restricted hotspots which do not necessarily cover 17 % of the territory or, on the contrary, additional sites which were threatened but not identified due to the 17 % threshold. Expected PD*loss* alternative hotspots were defined as areas where Expected PD*loss* was higher than the mean value of all sites and areas where Expected PD*loss* was higher than under a random distribution of threats (F_Expected PDloss_ ≥ 0.5). HEDGE and BED alternative hotspots were defined as areas where HEDGE and BED, respectively, contained at least one species from the 10 % of species with the highest HEDGE and BED scores and areas where HEDGE and BED, respectively, was higher than under a random distribution of threats (F_HEDGE/BED_ ≥ 0.5).

To assess the extent to which the selected hotspots complemented one another, we examined the unique Expected PD*loss* values, and the sum of species HEDGE and BED values of combined hotspots (considering the pool of species occurring in at least one of the hotspots) and compared these values to those expected if the hotspots were randomly distributed. A p value was defined as the frequency with which random values were higher or equal than the observed value. Finally, to test whether a species richness approach would have identified similar hotspots [33], we used Spearman correlations to assess the correlations between species richness and PD, the richness in threatened species (i.e., species classified as critically endangered, endangered or vulnerable in the IUCN Red List) and either Expected PD*loss* or HEDGE, and the richness in range-restricted species (number of species in the top 10 % species with the smallest range size) and BED.

### Are hotspots for the conservation of Mediterranean evolutionary history well protected?

For each group and metric, we calculated the observed proportion of hotspots of threatened and endemic evolutionary history that were protected. We then randomly designated 17 % of the grid cells as hotspots and calculated the number of those simulated priority grid cells that were protected. Finally, we calculated the frequency (termed F_PA_) with which the observed proportion of priority grid cells that were protected was greater than that in simulations. We considered the conservation of hotspots to be more efficient than if randomly distributed when the F_PA_ value was greater than 0.75, as efficient when the F_PA_ value ranged between 0.25 and 0.75, and less efficient when the F_PA_ value was less than 0.25. Due to the low coverage of the Mediterranean basin by protected areas we considered that, if the proportion of protected hotspots was higher than in our null models in more than 75 % of the simulations, hotspots were well protected.

We performed the calculations for all of the protected areas first and then for only the categories I, II and IV of protected areas. Categories I, II and IV require a higher level of protection, and their management objectives are specifically dedicated to species protection, whereas other categories may focus on other aspects of conservation, such as the sustainable use of resources [26]. Other authors have not included category IV protected areas as their regulation may not be as stringent as that of categories I and II [50, 51]. However, we included them in the present study because category IV encompasses different designations in the Mediterranean Basin, some of which are highly regulated (e.g., natural reserves, national parks) [26]. Out of the 9093 protected areas, 1837 were of categories I, II or IV.

We repeated this method for two protection scenarios: a minimum protection scenario, in which a grid cell was considered protected if it intersected at least one protected area; and a strong protection scenario, in which a grid cell had to have more than 50 % of its area covered by protected areas to be considered protected [70]. The latter scenario did not include protected areas for which range size information was missing (see Data section).

## Additional files

10.1186/s12898-016-0099-3 Identification of priority sites independently of Aïchi targets in terrestrial mammals. Data. A. Identification of Expected PD*loss* priority sites independently of Aïchi targets. Expected PD*loss* hotspots were defined as areas where Expected PD*loss* was higher than the mean value of all sites and areas where Expected PD*loss* was higher than under a random distribution of threats (F_Expected PDloss_ ≥ 0.5). B and C. Identification of HEDGE and BED priority sites independently of Aïchi targets: HEDGE and BED hotspots were defined as areas where HEDGE and BED, respectively, contained at least one species from the 10 % of species with the highest HEDGE and BED scores and areas where HEDGE and BED, respectively, was higher than under a random distribution of threats (F_HEDGE/BED_ ≥ 0.5).

10.1186/s12898-016-0099-3 Identification of priority sites independently of Aïchi targets in squamates. Data. A. Identification of Expected PD*loss* priority sites independently of Aïchi targets. Expected PDloss hotspots were defined as areas where Expected PDloss was higher than the mean value of all sites and areas where Expected PDloss was higher than under a random distribution of threats (F_Expected PDloss_ ≥ 0.5). B and C. Identification of HEDGE and BED priority sites independently of Aïchi targets: HEDGE and BED hotspots were defined as areas where HEDGE and BED, respectively, contained at least one species from the 10 % of species with the highest HEDGE and BED scores and areas where HEDGE and BED, respectively, was higher than under a random distribution of threats (F_HEDGE/BED_ ≥ 0.5).

10.1186/s12898-016-0099-3 Identification of priority sites independently of Aïchi targets in amphibians. Data: A. Identification of Expected PD*loss* priority sites independently of Aïchi targets. Expected PD*loss* hotspots were defined as areas where Expected PD*loss* was higher than the mean value of all sites and areas where Expected PD*loss* was higher than under a random distribution of threats (F_Expected PDloss_ ≥ 0.5). B and C. Identification of HEDGE and BED priority sites independently of Aïchi targets: HEDGE and BED hotspots were defined as areas where HEDGE and BED, respectively, contained at least one species from the 10 % of species with the highest HEDGE and BED scores and areas where HEDGE and BED, respectively, was higher than under a random distribution of threats (F_HEDGE/BED_ ≥ 0.5).

10.1186/s12898-016-0099-3 Spatial distribution of species richness in mammals. A. Species richness. B. Threatened species richness (number of species whose threat status is CR, EN or VU). C. Endemic species richness (number of species among the 10 % of species with the smallest range).

10.1186/s12898-016-0099-3 Spatial distribution of species richness in squamates. A. Species richness. B. Threatened species richness (number of species whose threat status is CR, EN or VU). C. Endemic species richness (number of species among the 10 % of species with the smallest range).

10.1186/s12898-016-0099-3 Spatial distribution of species richness in amphibians. A. Species richness. B. Threatened species richness (number of species whose threat status is CR, EN or VU). C. Endemic species richness (number of species among the 10 % of species with the smallest range).

10.1186/s12898-016-0099-3 conservation scenarios. Each graph represents the proportion of HEDGE, BED and Expected PD*loss* priority sites protected. Red bars correspond to the degree of protection if all protected areas are included and blue bars the degree of protection if only protected areas of categories I, II and IV are included. Star symbols correspond to the frequency to which the proportion of hotspots protected was higher than if priority grid cells were distributed randomly (F_PA_): no star means F_PA_ ≤ 0.25; * means 0.25 < F_PA_ ≤ 0.75; ** 0.75 < F_PA_ ≤ 1. We ran analysis for **A.** a scenario of minimum protection in which hotspots were safe if they intersected at least one protected area; **B.** a scenario of strong protection where a site was considered safe if it was protected on more than half of its area.

10.1186/s12898-016-0099-3 Justification of the data used.

10.1186/s12898-016-0099-3 Species not found in any protected areas and their respective HEDGE and BED scores. Data: Species not found in any protected areas of category I, II and IV, a * means that the species is not present in any protected area.

10.1186/s12898-016-0099-3 Data-deficient species and their BED scores. Data-deficient mammal and squamate species and their BED scores (there are no amphibian DD species in the Mediterranean basin).

10.1186/s12898-016-0099-3 HEDGE and BED scores of all Mediterranean species. HEDGE and BED scores of all Mediterranean species.

## Abbreviations

BED: biogeographic weighted evolutionary distinctiveness
ED: evolutionary distinctiveness
DD: data-deficient
Expected PD*loss*: expected loss of phylogenetic diversity
HEDGE: heightened evolutionary distinctiveness and global endangerment
IUCN: international union for conservation of nature
PD: phylogenetic diversity
