# Supplementary material for: Loss and conservation of evolutionary history in the Mediterranean Basin
Source: BMC Ecol. 2016 Oct 7;16:43. doi: 10.1186/s12898-016-0099-3 (PMC5055673; doi:10.1186/s12898-016-0099-3)
Supplement: Supplementary file 1 — 10.1186/s12898-016-0099-3 Identification of priority sites independently of Aïchi targets in terrestrial mammals. Data. A. Identification of Expected PDloss priority sites independently of Aïchi targets. Expected PDloss hotspots were defined as areas where Expected PDloss was higher than the mean value of all sites and areas where Expected PDloss was higher than under a random distribution of threats (FExpected PDloss ≥ 0.5). B and C. Identification of HEDGE and BED priority sites independently of Aïchi targets: HEDGE and BED hotspots were defined as areas where HEDGE and BED, respectively, contained at least one species from the 10 % of species with the highest HEDGE and BED scores and areas where HEDGE and BED, respectively, was higher than under a random distribution of threats (FHEDGE/BED ≥ 0.5). [file 12898_2016_99_MOESM1_ESM.pdf]

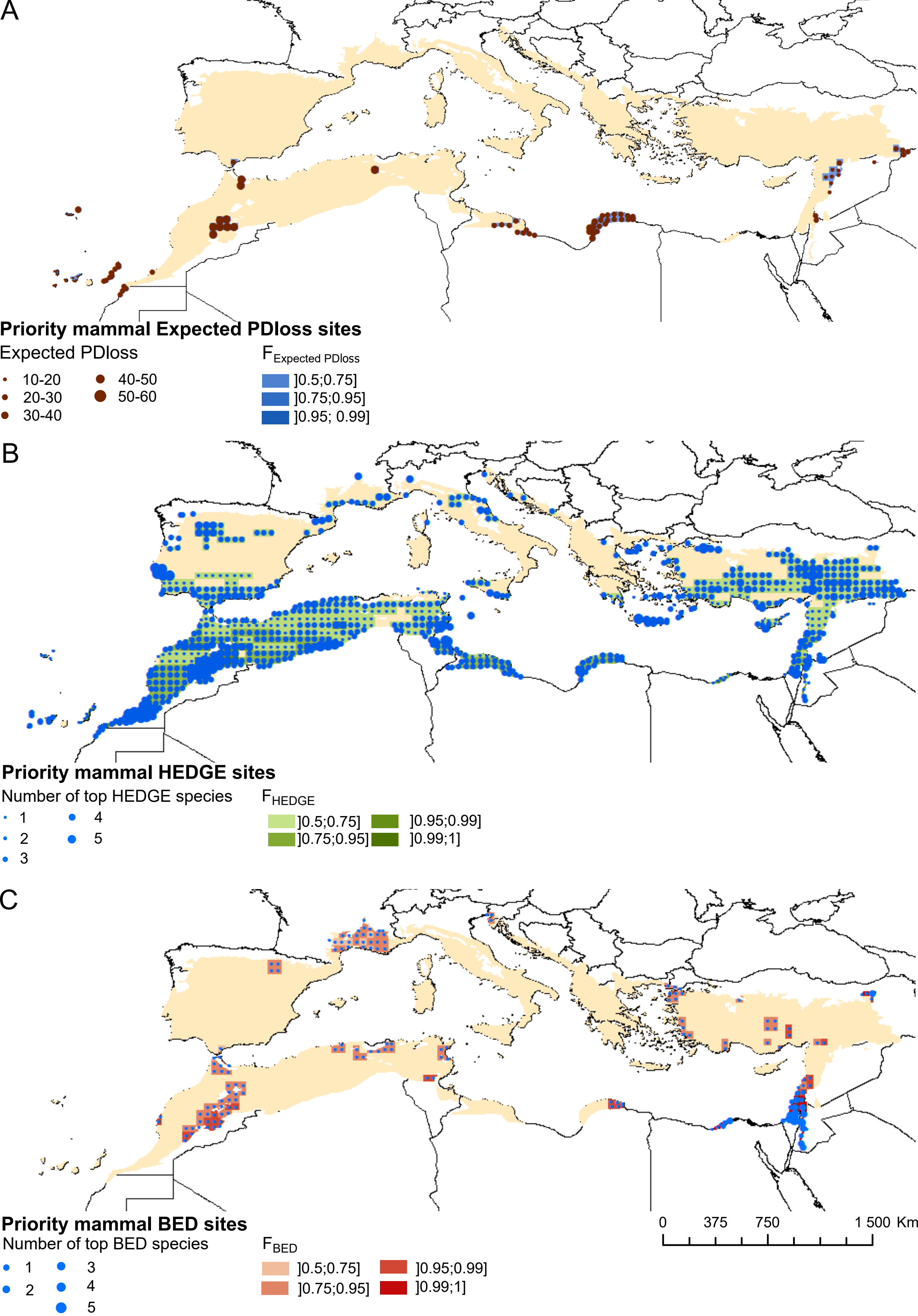
A
Priority mammal Expected PDloss sites
Expected PDloss
10-20
20-30
30-40
40-50
50-60
F Expected PDloss
]0.5;0.75]
]0.75;0.95]
]0.95; 0.99]
B
Priority mammal HEDGE sites
Number of top HEDGE species
1
2
3
4
5
F HEDGE
]0.5;0.75]
]0.75;0.95]
]0.95;0.99]
]0.99;1]
C
Priority mammal BED sites
Number of top BED species
1
2
3
4
5
F BED
]0.5;0.75]
]0.75;0.95]
]0.95;0.99]
]0.99;1]
0
375
750
1 500 Km
