# Supplementary material for: Loss and conservation of evolutionary history in the Mediterranean Basin
Source: BMC Ecol. 2016 Oct 7;16:43. doi: 10.1186/s12898-016-0099-3 (PMC5055673; doi:10.1186/s12898-016-0099-3)
Supplement: Supplementary file 5 — 10.1186/s12898-016-0099-3 Spatial distribution of species richness in squamates. A. Species richness. B. Threatened species richness (number of species whose threat status is CR, EN or VU). C. Endemic species richness (number of species among the 10 % of species with the smallest range). [file 12898_2016_99_MOESM5_ESM.pdf]

B. Squamates

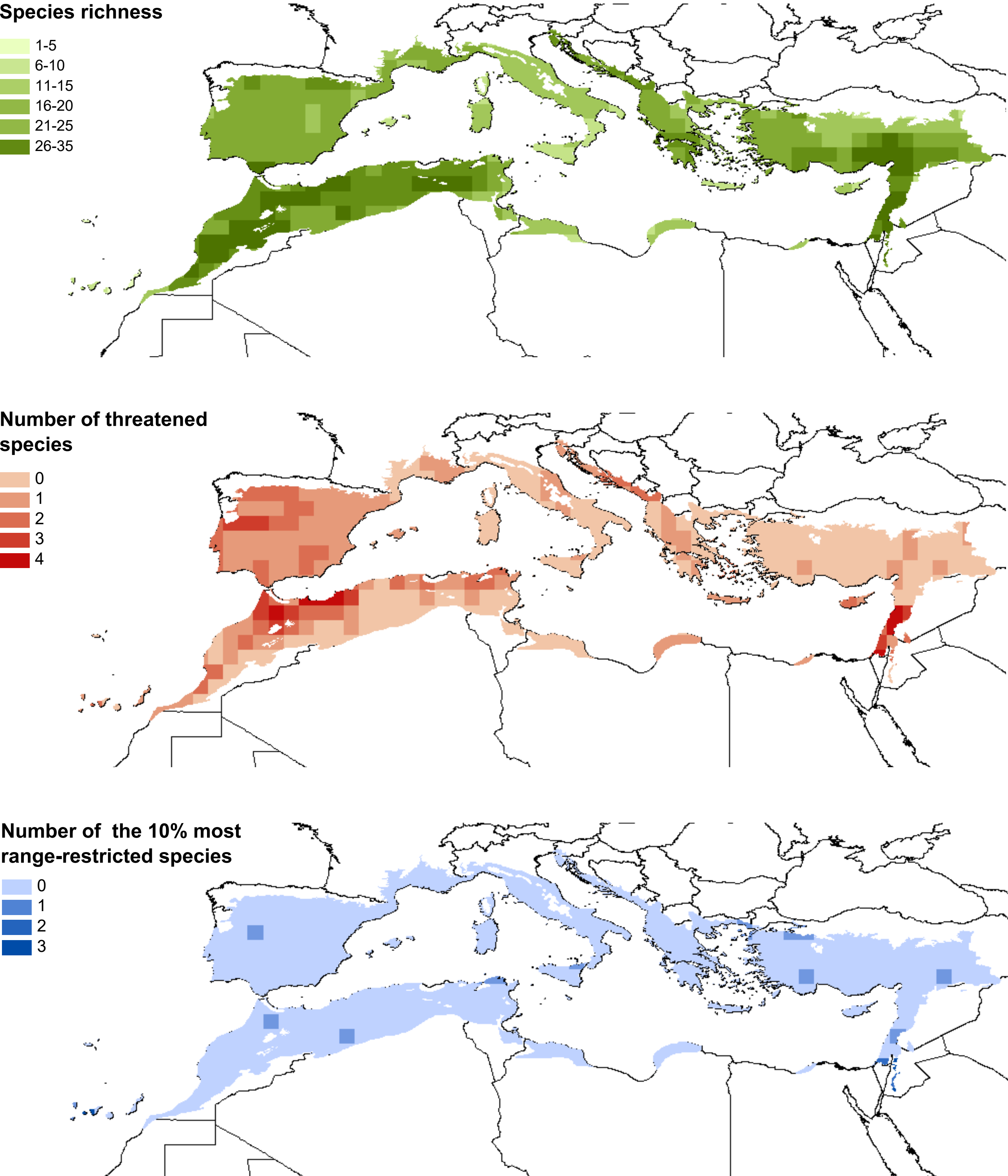

Species richness
1-5
6-10
11-15
16-20
21-25
26-35
Number of threatened species
0
1
2
3
4
Number of the 10% most range-restricted species
0
1
2
3
