# Supplementary material for: Loss and conservation of evolutionary history in the Mediterranean Basin
Source: BMC Ecol. 2016 Oct 7;16:43. doi: 10.1186/s12898-016-0099-3 (PMC5055673; doi:10.1186/s12898-016-0099-3)

## C. Amphibians

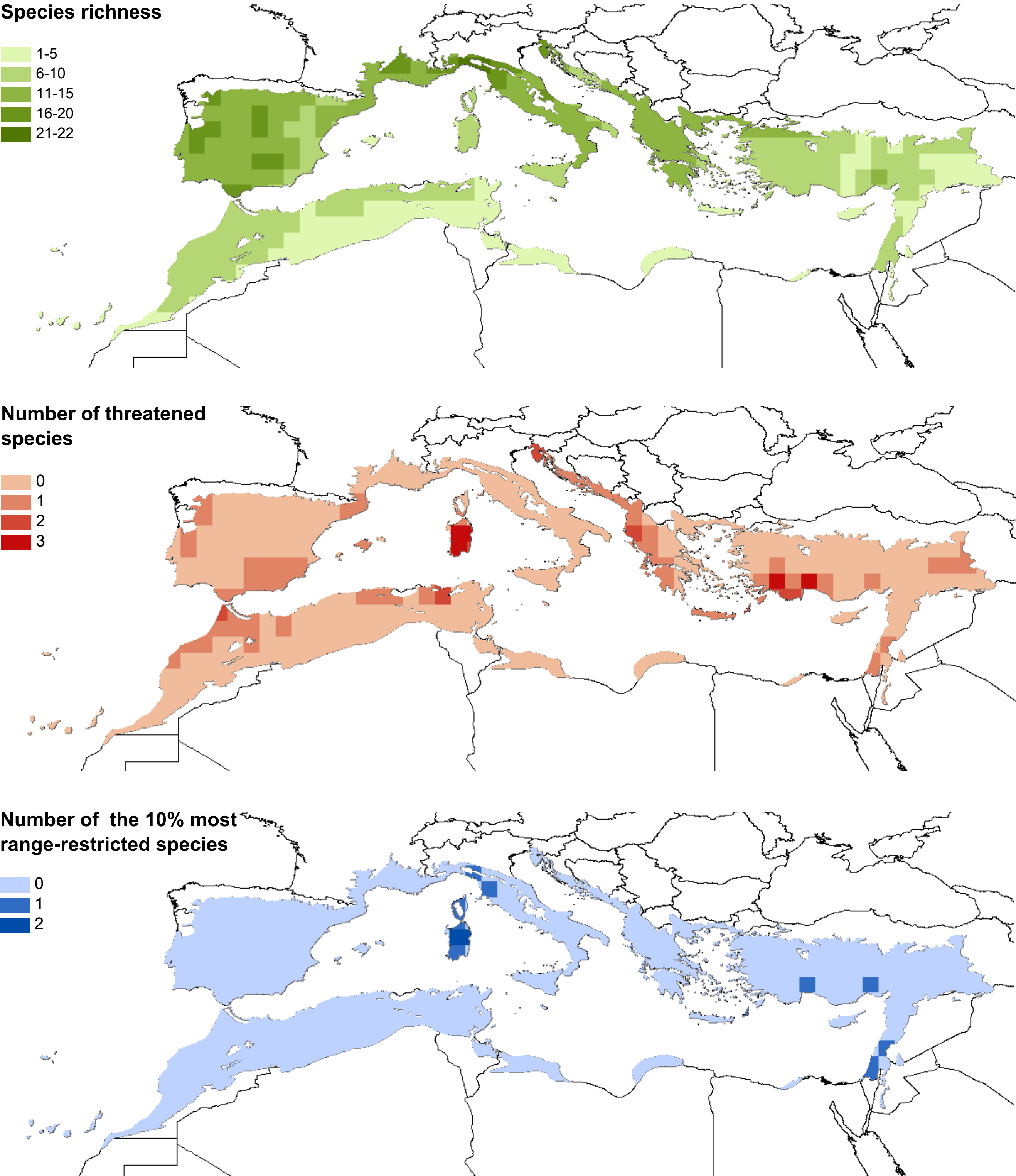

Supplement: Supplementary file 6 — 10.1186/s12898-016-0099-3 Spatial distribution of species richness in amphibians. A. Species richness. B. Threatened species richness (number of species whose threat status is CR, EN or VU). C. Endemic species richness (number of species among the 10 % of species with the smallest range). [file 12898_2016_99_MOESM6_ESM.pdf]
