# Supplementary figures and images for: Loss and conservation of evolutionary history in the Mediterranean Basin
Source: BMC Ecol. 2016 Oct 7;16:43. doi: 10.1186/s12898-016-0099-3 (PMC5055673; doi:10.1186/s12898-016-0099-3)

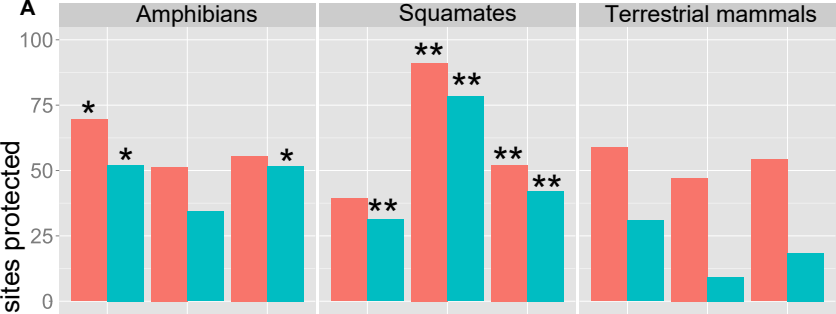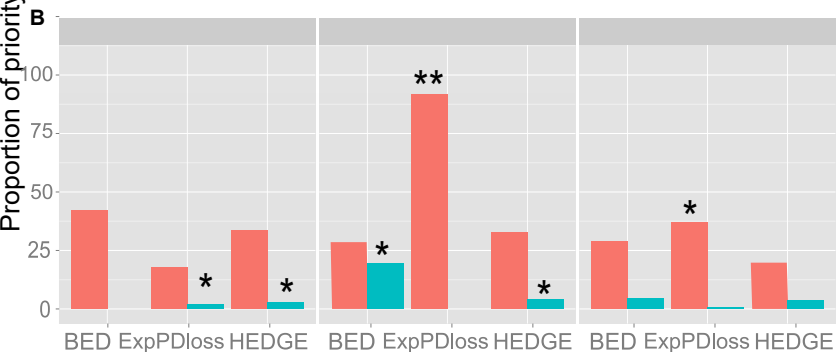

**Level of protection**

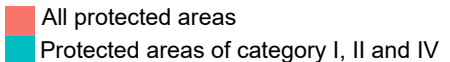

Supplement: Supplementary file 7 — 10.1186/s12898-016-0099-3 conservation scenarios. Each graph represents the proportion of HEDGE, BED and Expected PDloss priority sites protected. Red bars correspond to the degree of protection if all protected areas are included and blue bars the degree of protection if only protected areas of categories I, II and IV are included. Star symbols correspond to the frequency to which the proportion of hotspots protected was higher than if priority grid cells were distributed randomly (FPA): no star means FPA ≤ 0.25; * means 0.25 < FPA ≤ 0.75; ** 0.75 < FPA ≤ 1. We ran analysis for A. a scenario of minimum protection in which hotspots were safe if they intersected at least one protected area; B. a scenario of strong protection where a site was considered safe if it was protected on more than half of its area. [file 12898_2016_99_MOESM7_ESM.pdf]
