# Supplementary material for: Loss and conservation of evolutionary history in the Mediterranean Basin
Source: BMC Ecol. 2016 Oct 7;16:43. doi: 10.1186/s12898-016-0099-3 (PMC5055673; doi:10.1186/s12898-016-0099-3)
Supplement: Supplementary file 8 — 10.1186/s12898-016-0099-3 Justification of the data used. [file 12898_2016_99_MOESM8_ESM.docx]

**Additional file 8: Justification of the data used**

342 squamate species have been counted in the Mediterranean Basin ("Mediterranean forests, woodlands, and scrub" of the Paleartic Realm). We found range data from the IUCN global assessment for 238 of them. Among the 238 species, 26 species were absent from the phylogeny of Pyron & Burbrink (2014). Each of the 26 species were added in this global phylogeny at a node supporting species from the same genus. This created polytomies (nodes with 3 species) in the global phylogeny. Yet, ten out of the 26 species were the only representant of their genus in the Mediterranean Basin and one species had only one other species belonging to the same genus. Thus when the global phylogeny was pruned to consider only Mediterranean squamates only 15 polytomies remained. All species included through polytomies were least-concerned except Platyceps sinai (nearly threatened) and Vipera anatolica (critically endangered) in the IUCN Red List. Among the 238 reptile species, 8 were data-deficient in the IUCN Red List. The 238 species were thus considered for BED assessments and 230 only for HEDGE and ExpPDloss assessments because HEDGE and ExpPDloss indices need information about threat status but not the BED index.

107 amphibian species have been observed in the Mediterranean Basin. (Cox et al. 2006). Among them, 8 species were included at a node supporting species from the same genus in the global phylogeny of Pyron & Wiens (2013). Three species were the only representants of their genus in the Mediterranean basin, thus the phylogeny of Mediterranean amphibians contained 5 polytomies. All the 107 species had sufficient data for a classification in the IUCN Red List (from least concerned to critically endangered).

280 mammals were observed in the Mediteranean Basin ("Mediterranean forests, woodlands, and scrub" of the Paleartic Realm) and we compiled spatial data for all of them. We removed 8 marine species (including *Monachus monachus*). We also removed 11 NA species and 3 regionally extinct species. Fifteen of the remaining species were not present in the phylogeny of Rolland et al. (2014) and we added them as polytomies at a node supporting species from the same genus. Among them, one was endangered (*Ovis orientalis*), two were vulnerable (*Plecotus sardus* and *Capra aegagrus*) and the others were data-deficient (6 species) or Least-Concern (6 species). Moreover there was 29 terrestrial DD species (regional status) in the Mediterranean basin. We thus compiled 258 species for the BED analysis and 229 species for the HEDGE and ExpPDloss analysis
